# Thermal, Structural, and Morphological Analysis of ZnFe_2_O_4_ Embedded and Non-Embedded in a SiO_2_ Matrix for Magnetic and Photocatalytic Applications

**DOI:** 10.3390/nano15211644

**Published:** 2025-10-28

**Authors:** Thomas Dippong, Anamaria-Magdalena Savolszki-Madaras, Raul Marius Reiz, Ioan Petean, Oana Cadar

**Affiliations:** 1 Faculty of Science, Technical University of Cluj-Napoca, 76 Victoriei Street, 430122 Baia Mare, Romania; 2 Faculty of Chemistry and Chemical Engineering, Babes-Bolyai University, 11 Arany Janos Street, 400084 Cluj-Napoca, Romania; ioan.petean@ubbcluj.ro; 3 National Institute for Research and Development of Optoelectronics INOE 2000, Research Institute for Analytical Instrumentation, 67 Donath Street, 400293 Cluj-Napoca, Romania; oana.cadar@icia.ro

**Keywords:** nanoparticles, sol-gel, crystallinity, morphology, VSM, photocatalysis

## Abstract

This study compares the structural, morphological, magnetic, and photocatalytic properties of a pure SiO_2_ matrix, a ZnFe_2_O_4_-doped SiO_2_ nanocomposite (both synthesized via the sol-gel method), and bulk ZnFe_2_O_4_ produced by thermal decomposition. Thermogravimetric analysis (TGA) reveals that metal oxalates form below 200 °C and decompose into metal oxides, which subsequently form ferrite. Fourier-transform infrared (FTIR) spectroscopy confirms the embedding of both undoped and ZnFe_2_O_4_-doped nanoparticles into the SiO_2_ matrix at all investigated annealing temperatures. X-ray diffraction (XRD) consistently reveals the formation of crystalline ZnFe_2_O_4_, with the crystallite size increasing from 48 to 93 nm upon annealing. Atomic force microscopy (AFM) shows spherical ferrite nanoparticles surrounded by an amorphous layer, with particle growth observed at higher temperatures. Structural parameters derived from XRD (e.g., crystallite size, density, porosity, lattice constant, unit cell volume) and AFM (e.g., particle size, coating thickness) as well as magnetic parameters (saturation magnetization, remanence, anisotropy, coercivity) demonstrate clear dependence on both dopant presence and annealing temperature. Magnetic measurements reveal enhanced properties with increasing ferrite content and heat treatment, with a transition from superparamagnetic behavior at 700 °C to ferrimagnetic behavior above 1000 °C. Scavenger experiments confirmed the involvement of holes, hydroxyl radicals, and superoxide radicals in the photocatalytic process. The photocatalytic efficiency, as evaluated by the Rhodamine B degradation under visible light, highlights the promising potential of the obtained nanocomposite for advanced environmental and technological applications.

## 1. Introduction

Nanostructured ferrites exhibit outstanding magnetic properties, including high saturation magnetization (*Ms*), low coercivity (*Hc*), high permeability, large specific surface area, high electrical resistivity, low eddy current and dielectric losses, as well as mechanical hardness, chemical stability, physical flexibility, and biocompatibility [1]. These attributes make them suitable for a broad range of applications, including environmental remediation [2], biomedical uses (e.g., anticancer, antibacterial, bioimaging, and biosensors) [3,4], energy storage, spintronics, ferrofluids, imaging and therapy, electronics, high-frequency devices, and military technologies [3,4,5,6]. Their chemical and thermal stability, mechanical robustness [7], non-toxicity [8], corrosion resistance, and low cost further enhance their applicability. However, the structural and magnetic properties of ferrites are highly sensitive to factors such as chemical composition, precursor type, synthesis route, thermal treatment conditions, and the presence of dopants, impurities, or secondary phases [4,5,6,7,8,9,10,11,12].

Cubic ferrites, with the general formula MFe_2_O_4_ (where M = Co, Cu, Mg, Mn, Ni, Zn, or their combinations), are soft magnetic materials that crystallize in a spinel structure in which M^2+^ ions typically occupy tetrahedral (A) sites and Fe^3+^ reside in octahedral (B) sites [4,7,9]. The cation distribution plays a crucial role in determining the structural, magnetic, and electronic properties of these materials [7]. Zinc ferrite (ZnFe_2_O_4_) adopts a normal spinel structure, with Zn^2+^ ions occupying A- sites and Fe^3+^ ions residing in B- sites [3]. In ceramics with well-developed porosity, such as nanostructured MgAl_2_O_4_ spinel, positron annihilation occurs through the positron trapping channel and ortho-positronium decay. This approach can be applied to a wide range of porous functional nanomaterials [13].

The synthesis of uniform nanostructured ferrite particles is critical, as particle size significantly influences their optical, electrical, and magnetic properties [10,11,12]. These properties are strongly affected by factors such as the synthesis method, cation stoichiometry, type of divalent cations, and heat treatment conditions [7,14]. Various techniques are employed to prepare nanoferrites, including sol-gel, heat treatment temperature, hydrothermal, coprecipitation, sonochemical processing, dry and wet grinding, and microemulsion methods [15]. Among these, heat treatment temperature plays a pivotal role in microstructural evolution, namely lower temperatures typically result in higher porosity and limited densification, whereas higher temperatures promote grain growth and improved densification [11,16,17,18]. The sol-gel method offers several advantages, including rapid reaction kinetics, high chemical homogeneity, cost-effectiveness, energy efficiency, compositional flexibility, low processing temperatures, monodisperse particle distribution, and well-controlled surface morphologies [7,19,20].

Embedding ferrites in a SiO_2_ matrix further improves material performance by reducing nanoparticle agglomeration, as SiO_2_ acts as a chemically stable, non-toxic, inert spacer and protective coating owing to its network-forming ability [11,17,18]. Although SiO_2_ is non-magnetic, it can indirectly affect the magnetic and photocatalytic behavior of ferrites by modifying their structure and morphology [16,17,18,21]. SiO_2_ attends as a versatile component in photocatalytic materials, enhancing surface properties, charge separation, and stability in order to boost efficiency [22,23]. For example, in TiO_2_/SiO_2_ composites, SiO_2_ improves TiO_2_ dispersion, stability, and surface area, facilitating contaminant adsorption [22]. In ZnFe_2_O_4_/SiO_2_ nanocomposites, SiO_2_ prevents nanoparticle agglomeration, increases the specific surface area (SSA), and enhances structural and thermal stability, thereby improving photocatalytic performance [22,23]. In g-C_3_N_4_/SiO_2_ composites, SiO_2_ increases SSA, enabling contaminant adsorption and providing more reactive sites. It also promotes electron–hole pair separation and migration, reducing charge recombination and improving photocatalytic efficiency [22].

ZnFe_2_O_4_ nanoparticles have demonstrated effective catalytic activity in the degradation of organic dyes and the removal of toxic metals, dyes, and organic contaminants, including methylene blue, Direct Red 81, Evans blue, and Malachite green [2]. Their magnetic properties enable efficient recovery and reuse, supporting their applicability in large-scale catalytic and depollution processes [10]. The effectiveness of ZnFe_2_O_4_ as a photocatalyst and nanosorbent is attributed to its regenerability, versatility, and facile separation, maintaining high photocatalytic performance over at least five consecutive cycles without significant loss of activity [2,24]. Photocatalytic efficiency is strongly influenced by surface area, particle size, and dopant concentration [1]. Due to their narrow band gaps, ZnFe_2_O_4_ nanoparticles are particularly advantageous for visible-light-driven applications, such as pollutant degradation in wastewater treatment, where performance is closely linked to morphological features such as surface area and particle size [25]. The magnetic properties of nanostructured ferrites depend on several factors, including particle size, synthesis route, surface energy, and thermal treatment conditions [7,26]. In ferrites, ferromagnetism arises from Fe^3+^ ions occupying both A- and B- sites, enabling strong superexchange interactions, which tend to weaken as particle size increases. ZnFe_2_O_4_ typically exhibits paramagnetic behavior and relatively high coercivity at room temperature [26].

The objectives of this study are (i) to compare the thermal decomposition and sol-gel methods for synthesizing ZnFe_2_O_4_, both embedded and non-embedded in a SiO_2_ matrix; (ii) to investigate thermal transformation processes and the formation of succinate precursors via thermal analysis; (iii) to perform structural and morphological characterization using Fourier-transform infrared spectroscopy (FT-IR), X-ray diffraction (XRD), and atomic force microscopy (AFM), with particular emphasis on matrix formation and its influence on the ferrite structure; (iv) to synthesize ZnFe_2_O_4_ nanoparticles via a modified sol-gel method and evaluate their photocatalytic performance in degrading Rhodamine B; and (v) to analyze the magnetic properties (saturation magnetization (*M_S_*), remanence magnetization (*M_R_*), coercivity (*H_C_*), and anisotropy (*K*)) as a function of the SiO_2_ matrix and thermal temperature.

## 2. Materials and Methods

### 2.1. Synthesis

**Figure 1:**
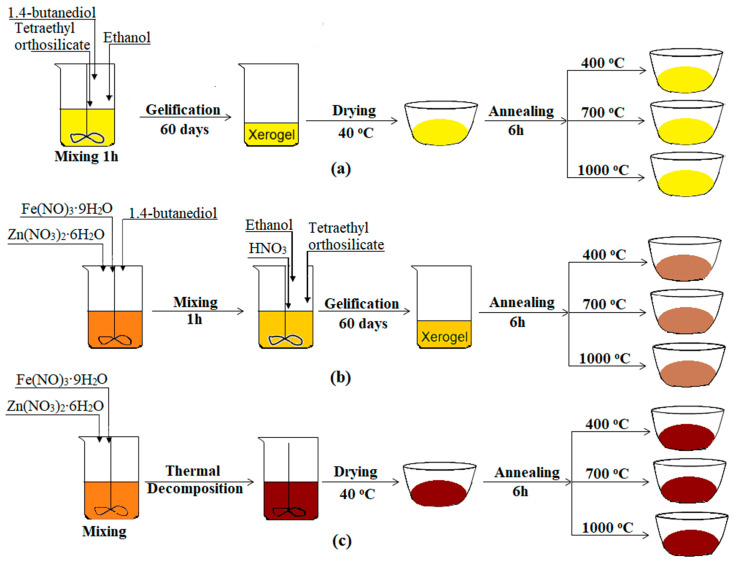
Flowchart of the synthesis for SiO_2_ (**a**), (ZnFe_2_O_4_)_0.5_/(SiO_2_)_0.5_ (**b**), and ZnFe_2_O_4_ (**c**).

SiO_2_ and 50%ZnFe_2_O_4_/50%SiO_2_ samples were synthesized via a modified sol-gel method using zinc nitrate hexahydrate (Zn(NO_3_)_2_·6H_2_O), iron nitrate nonahydrate (Fe(NO_3_)_3_·9H_2_O), tetraethyl orthosilicate (TEOS), 1,4-butanediol, and ethanol. The synthesis involved the dropwise addition of an ethanolic TEOS solution to a nitric acid-acidified diol–ethanol mixture under vigorous magnetic stirring at room temperature. The resulting clear solution was stirred for an additional 30 min and then allowed to gel under ambient conditions. After gelation, the xerogels were crushed and dried at 40 °C for 6 h. Thermal treatments were conducted at 200, 400, 700, and 1000 °C for 6 h, with a heating rate of 20 °C/min, using an LT9 muffle furnace (Nabertherm, Lilienthal, Germany) (Figure 1a,b).

The ZnFe_2_O_4_ sample was synthesized via thermal decomposition of Zn(II) and Fe(III) succinate precursors, which were obtained through a redox reaction among the corresponding metal nitrates and 1,4-butanediol. The resulting material was dried at 40 °C for 6 h, followed by thermal decomposition at 200 °C for 6 h. Subsequent annealing was performed at 400, 700, and 1000 °C for 6 h, with a heating rate of 20 °C/min, in an LT9 muffle furnace (Nabertherm) (Figure 1c).

### 2.2. Characterization

The formation and decomposition of carboxylation precursors were monitored by thermogravimetric (TG) and differential thermal (DTA) analyses in air up to 1000 °C at a heating rate of 5 °C·min^−1^ using alumina standards and an SDT Q600 (TA Instruments, New Castle, DE, USA).

FT-IR spectra were recorded from 1% KBr pellets using a Prestige-21 (Shimadzu, Tokyo, Japan).

The XRD measurements were conducted at room temperature using a D8 Advance diffractometer (Bruker, Karlsruhe, Germany) employing a CuK_α1_, radiation source (λ = 1.5418 Å) operated at 40 kV and 40 mA.

The specific surface area (SSA) was determined at liquid nitrogen temperature (−196 °C) using a Sorptomatic 1990 (Thermo Scientific, Waltham, MA, USA) analyzer. Prior to the measurements, the samples were degassed at 200 °C under a vacuum pressure of 2 Pa for 4 h.

For AFM, thin films were prepared by dispersing 1 g of powder in 10 mL deionized water and stirring magnetically for 15 min. Clean glass slides were vertically immersed in the suspension for 30 s, then slowly withdrawn, and dried horizontally at room temperature to allow for the self-assembly of nanoparticles. The films were characterized using a JSPM-4210 scanning probe microscope (JEOL, Tokyo, Japan) in AC mode, employing NSC15-Hard probes (MikroMasch, Sofia, Bulgaria) at a resonant frequency of 325 kHz and a spring constant of 40 nN/nm over a 1 µm^2^ scan area. Topographic images were analyzed using WinSPM 2.0 software (JEOL) to assess surface roughness and particle size. Topographic images were analyzed using WinSPM 2.0 software (JEOL) to assess surface roughness and particle size. At least three different macroscopic sites were investigated, and the mean values were provided with the standard deviation (±).

SEM microscopy was effectuated with a Hitachi SU8230 (Hitachi Company, Tokyo, Japan) operated in high vacuum mode at an acceleration voltage of 30 kV. TEM microscopy was effectuated with an HD-2700 (Hitachi, Tokyo, Japan) on the dispersed ferrite powder samples. Electron microscopy images were analyzed with the professional software Image J 1.53t (National Institute of Health, Bethesda, Rockville, MD, USA), assessing the pores and particle distribution.

Magnetic measurements were conducted using a CFM-12 T cryogen-free VSM magnetometer (Cryogenic Ltd., London, UK). *M_S_* was determined under magnetic fields up to 10 T, and hysteresis loops were recorded between −2 T and 2 T at 300 K. To prevent particle movement during measurements, powder samples were embedded in epoxy resin.

The photocatalytic performance was assessed via Rhodamine B (RhB) degradation under visible light in a laboratory-scale reactor equipped with a 400 W halogen lamp (Osram, Premstätten, Austria) and an ultrasonic bath. To investigate the active species generated in the photocatalytic system (PS) consisting of a photocatalyst, RhB, and H_2_O_2_, scavenger experiments were performed. Ethylenediaminetetraacetic acid (EDTA-2Na, 10 mM), benzoquinone (p-BQ, 1 mM), AgNO_3_ (100 mM), and tert-butyl alcohol (t-BuOH) (1:20 vol.) were used as scavengers introduced into the PS in the amount of 1 mL to capture holes (h•), superoxide radicals (•O_2_^−^), electrons (e^−^), and hydroxyl radicals (OH•), respectively. Tests of cyclic photocatalysis were carried out using TiO_2_@0.2%Fe_2_O_3_ and TiO_2_@2%Fe_2_O_3_ heterostructures. After dark adsorption, a 90 min photocatalytic decomposition process of RhB was carried out and repeated four times. After each decomposition process, the photocatalyst was separated from the solution of RhB by centrifugation and washed with ethanol three times. After that, the powder was dried for 4 h at 70 °C and used again.

## 3. Results and Discussions

### 3.1. Thermal Analysis

**Figure 2:**
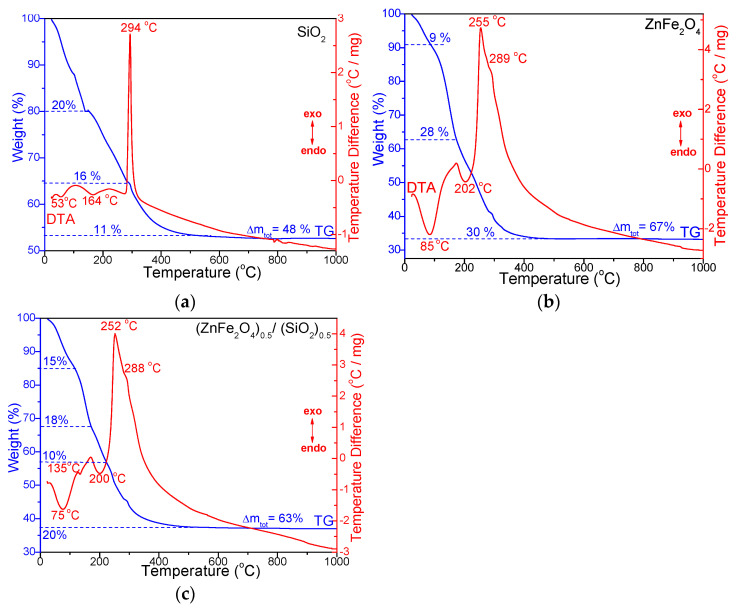
TG and DTA curves of SiO_2_ (**a**), 50%ZnFe_2_O_4_/50%SiO_2_ (**b**), and ZnFe_2_O_4_ (**c**) dried at 40 °C.

Figure 2 shows the TG–DTA curves of (a) SiO_2_, (b) 50%ZnFe_2_O_4_/50%SiO_2_, and (c) ZnFe_2_O_4_ non-embedded in SiO_2_. The SiO_2_ sample exhibits two endothermic effects at 53 °C and 164 °C, corresponding to a ~20% mass loss, attributed to the removal of adsorbed water, condensation reactions, and the elimination of unbound 1,4-butanediol [18,27,28]. A pronounced exothermic peak at 294 °C indicates the combustion of organic chains chemically bound into the SiO_2_ matrix [18,27]. The bulk ZnFe_2_O_4_ sample decomposes in four distinct steps: (*i*) an endothermic peak at 85 °C (~9% mass loss), associated with the release of crystallization water and residual moisture; (*ii*) an endothermic peak at 202 °C (~28% mass loss), associated with the formation of Zn^2+^ and Fe^3+^ succinates via a redox reaction between the corresponding nitrates and 1,4-butanediol; and (*iii*) two successive exothermic peaks at 255 °C and 289 °C (~30% total mass loss), corresponding to the decomposition of Zn^2+^ and Fe^3+^ succinates into amorphous ZnO and Fe_2_O_3_. The intermediate oxides subsequently react in situ to form the ZnFe_2_O_4_ spinel phase [16,17,18,27,28].

The DTA curve of the 50%ZnFe_2_O_4_/50%SiO_2_ gels dried at 40 °C (Figure 2) reveals the following thermal effects: (*i*) an endothermic peak at 75 °C with ~15% mass loss, attributed to the removal of residual moisture and physically adsorbed water; (*ii*) two additional endothermic peaks at 135 °C and 200 °C, corresponding to the formation of Zn^2+^ and Fe^3+^ succinates, with associated mass losses of 18% and 10%. This process is driven by the higher acidity of [Fe(H_2_O)_6_]^3+^ compared to [Zn(H_2_O)_6_]^2+^, which facilitates redox reactions between metal nitrates and 1,4-butanediol, accompanied by the evolution of volatile products (H_2_O, NO_2_) [16,17,18,27,28]; and (*iii*) two exothermic peaks at 252 °C and 288 °C (~20% mass loss), indicating the oxidative decomposition of metal succinates into ZnO and Fe_2_O_3_, followed by in situ formation of ZnFe_2_O_4_ spinel.

The total mass loss was highest for bulk ZnFe_2_O_4_ (67%), slightly lower for the ZnFe_2_O_4_/SiO_2_ composite (63%), and lowest for pure SiO_2_ (48%). The thermal behavior of the composite reflects overlapping processes within the SiO_2_ matrix, which makes it difficult to unambiguously attribute the observed thermal effects solely to succinate decomposition [27].

### 3.2. FT-IR Analysis

**Figure 3:**
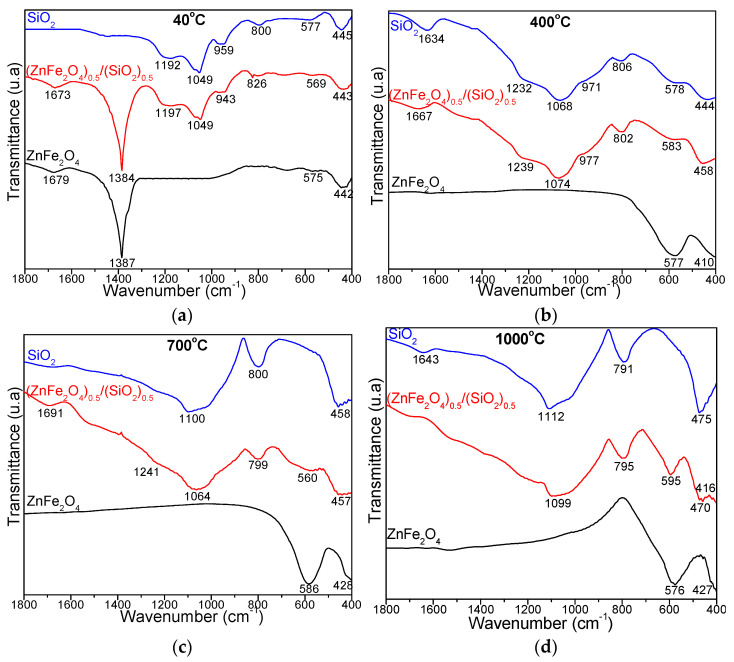
FT-IR spectra of SiO_2_, (ZnFe_2_O_4_)_0.5_/(SiO_2_)_0.5_ and ZnFe_2_O_4_ heat treated at 40 °C (**a**) and annealed at 400 °C (**b**), 700 °C (**c**), and 1000 °C (**d**).

Figure 3 displays the FT-IR spectra of the gels dried at 40 °C and 200 °C. All samples dried at 40 °C, except pure SiO_2_ (which lacks nitrate precursors), exhibit a characteristic nitrate band at 1384–1387 cm^−1^ [16,17,18,27,28]. This band disappears after heating to 200 °C, indicating complete nitrate decomposition of nitrates at this temperature. Absorption bands at 1673–1679 cm^−1^ correspond to the symmetric and asymmetric –OH stretching vibrations, originating from 1,4-butanediol and crystallization water in the metal nitrates. The formation of the SiO_2_ matrix in both the pure SiO_2_ and the 50% ZnFe_2_O_4_/50% SiO_2_ samples is confirmed by the presence of characteristic Si–O stretching bands at 1192–1216 cm^−1^, Si–O–Si stretching at 1049–1069 cm^−1^, Si–OH deformation at 943–955 cm^−1^, and Si–O chain vibrations within SiO_4_ tetrahedra at 799–826 cm^−1^ [16,17,18,27,28]. Bands observed at 569–582 cm^−1^ are assigned to tetrahedral M–O (metal–oxygen) stretching and cyclic Si–O–Si structures, while bands at 433–448 cm^−1^ correspond to octahedral M–O stretching and Si–O vibrations [16,17,18,27]. In bulk ZnFe_2_O_4_, bands at 572–575 cm^−1^ and 405–442 cm^−1^ are associated with Zn–O bending and Fe–O vibrations, respectively [16,17,18,27,28].

The formation of the SiO_2_ matrix in both the pure SiO_2_ and the 50% ZnFe_2_O_4_/50% SiO_2_ samples is confirmed at 400, 700, and 1000 °C by characteristic absorption bands. These bands correspond to symmetric stretching and bending vibrations of Si–O–Si chains (791–806 cm^−1^), in-plane and out-of-plane Si–O–Si stretching (1064–1112 cm^−1^), the Si–OH deformation (971–977 cm^−1^), the cyclic Si–O–Si structures (560–595 cm^−1^), and the Si–O bond vibrations (444–470 cm^−1^). A bending vibration of adsorbed H–O–H groups, attributable to atmospheric moisture, is observed at 1634–1691 cm^−1^. M–O vibrations are indicated by two intrinsic bands: Fe–O stretching vibrations in A-sites at 410–458 cm^−1^ and Zn–O vibrations in B-sites at 560–595 cm^−1^ [16,17,18,27,28].

### 3.3. XRD Analysis

**Figure 4:**
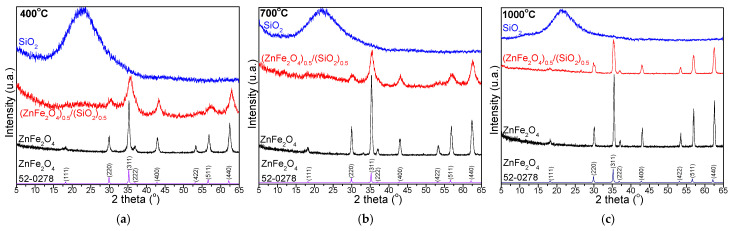
XRD patterns for ZnFe_2_O_4_, (ZnFe_2_O_4_)_0.5_/(SiO_2_)_0.5_, and SiO_2_ annealed at 400 (**a**), 700 (**b**), and 1000 ° C (**c**).

At all temperatures, the XRD pattern of SiO_2_ (Figure 4) displays a broad amorphous halo between 15 and 25° 2θ, characteristic of its amorphous structure, with no crystalline phases of the SiO_2_ matrix identified. At 400 °C (Figure 4a), the (ZnFe_2_O_4_)_0.5_/(SiO_2_)_0.5_ displays broad, low-intensity reflections corresponding to ZnFe_2_O_4_, indicating incomplete crystallization, reduced crystallite size, and the dilution effect caused by the amorphous SiO_2_ matrix. In contrast, pure ZnFe_2_O_4_ exhibits distinct diffraction peaks indexed to the (111), (220), (311), (400), and (511) planes, consistent with the spinel structure (JCPDS No. 52-0278) [28]. The sharper and more intense peaks observed in the pure phase suggest larger crystallite sizes compared to the ZnFe_2_O_4_ embedded in the SiO_2_ matrix.

At 700 °C (Figure 4b), the XRD pattern of (ZnFe_2_O_4_)_0.5_/(SiO_2_)_0.5_ shows ZnFe_2_O_4_ diffraction peaks that are clearer than those at 400 °C. However, the peaks remain broadened and of lower intensity, reflecting the influence of the amorphous SiO_2_ matrix and small crystallite size. In contrast, the pure ZnFe_2_O_4_ sample displays sharper and more intense peaks, indicating increased crystallinity and crystallite growth at this temperature.

At 1000 °C (Figure 4c), the XRD pattern of (ZnFe_2_O_4_)_0.5_/(SiO_2_)_0.5_ shows the characteristic diffraction peaks of pure ZnFe_2_O_4_, while the intensity remains low due to the dilution effect of the amorphous SiO_2_ matrix [28]. Continued peak broadening indicates limited crystallite growth and suggests that the inert SiO_2_ surface layer constrains grain coarsening.

The average crystallite size (D_CS_) was estimated by applying the Scherrer equation (Equation (1)) [16,17,18,27,28,29,30]:

(1) $$D_{CS}=\frac{0.9\cdot \lambda }{\beta \cdot cos\theta }$$

where λ is the wavelength of CuK_α_ radiation (1.5418 Å), β is the broadening of full width at half the maximum intensity (FWHM), and θ is the Bragg diffraction angle (°).

The D_C_s increases with increasing annealing temperature, ranging from 35 to 79 nm in the (ZnFe_2_O_4_)_0.5_/(SiO_2_)_0.5_ nanocomposite and from 48 to 95 nm in bulk ZnFe_2_O_4_. This behavior indicates that crystal growth is closely linked to the degree of cation disorder within the ferrite lattice, as incomplete cation occupancy can hinder grain coalescence. Furthermore, variations in cation valence states and ionic radii contribute to increased lattice strain and generate additional nucleation sites, promoting the formation of smaller crystallites [16,17,18,27]. Beyond thermal and instrumental contributions, the variation in D_Cs_ may also arise from peak broadening effects due to lattice strain and the physical constraint on grain growth imposed by the SiO_2_ matrix. Notably, D_CS_ plays a pivotal role in governing the magnetic properties of the nanocomposites, particularly when the grain size approaches the crystallite size [16,17,18,27].

The lattice constant, a (Å), was calculated from Bragg’s law with the Nelson–Riley function (Equation (2)) [16,17,18,27]:

(2) $$a=\frac{\lambda \sqrt{h^{2}+k^{2}+l^{2}}}{2\cdot sin\theta }$$

where λ is the wavelength of CuKα radiation (1.5406 Å), *hkl* are the Miller indices, and θ is the Bragg angle (°).

The lattice parameter (a) increases linearly from 8.345 to 8.411 Å with increasing Zn content, reflecting lattice expansion attributed to the larger ionic radius of Zn^2+^ (0.74 Å) compared to Fe^3+^ ions (0.64 Å (Table 1)). Deviations between experimental and theoretical lattice parameters arise from the simplifying assumption that ions behave as rigid spheres fixed within an ideal lattice. Zn^2+^ ions predominantly occupy the A- sites, whereas Fe^3+^ ions are distributed between both tetrahedral (A) and B- sites, depending on cation disorder and synthesis conditions [16,17,18,27,28,29,30].

The unit cell volume (V) and the X-ray density (d_XRD_), which reflect atomic packing within the crystal lattice, were derived from the XRD data using Avogadro’s number (6.022 · 10^23^ mol/L) and the ferrite molar mass according to Equations (3) and (4) [29,30]. Both a and V increase with annealing temperature, consistent with the trends observed in related ferrite systems [16,17,18,27,28,29,30]. The physical density (d_p_) was calculated according to the Archimedes’ principle by measuring the dry and suspended weights in xylene (Equation (5)), with lower d_p_ values indicative of increased porosity [18,27,28,29,30]:

(3) $$V=a^{3}$$

(4) $$d_{XRD}=\frac{8\cdot M_{w}}{{N_{A}\cdot a}^{3}}$$

(5) $$d_{p}=\frac{w\cdot \rho }{w-w^{\prime }}$$

where w is the sample weight in air (g), w′ is the sample weight in xylene (g), ρ is the xylene density (g/cm^3^), 8 is the quantity of molecules within the unit cell, Mw is the molecular weight, and N_A_ is Avogadro’s number (6.022 · 10^23^ mol^−1^).

Embedding ZnFe_2_O_4_ within the SiO_2_ matrix along with increasing the annealing temperature results in an increased V, ranging from 581 to 586 Å^3^ for the (ZnFe_2_O_4_)_0.5_/(SiO_2_)_0.5_ nanocomposite and 587–595 Å^3^ for bulk ZnFe_2_O_4_. This expansion is attributed to the generation of oxygen vacancies, a reduction in crystallization temperature, and an increase in molecular weight, which collectively outweigh the expected volumetric contraction during densification [16,17,18,27,28,29,30].

The X-ray density (d_XRD_) of the samples is calculated using Equation (4) [29,30]. Our results indicate that the d_XRD_ is highest in bulk ZnFe_2_O_4_ and decreases with increasing annealing temperature (5.512–5.465 g/cm^3^ for (ZnFe_2_O_4_)_0.5_/(SiO_2_)_0.5_, 5.456–5.383 g/cm^3^ for ZnFe_2_O_4_). This decrease is likely due to the formation of microstructural pores at higher temperatures [16,17,18,27].

The porosity (*P*) was evaluated using *dp* and *d_XRD_* using Equation (6) [15,16,17,24,25,26,27]:

(6) $$P=\left(1-\frac{d_{p}}{d_{XRD}}\right)100$$

The P values were higher in bulk ZnFe_2_O_4_ compared to ZnFe_2_O_4_ embedded in the SiO_2_ matrix, attributable to increased vacancy and pore formation during synthesis. Conversely, increased annealing temperatures promote densification, thereby reducing porosity (17.4–15.4% for (ZnFe_2_O_4_)_0.5_/(SiO_2_)_0.5_ and 19.6–19.0% for bulk ZnFe_2_O_4_). Low porosity is critical for achieving high-performance magnetic materials, as excess porosity degrades magnetic properties. The observed variations in P values are also influenced by the presence of Zn^2+^ ions, which generate additional oxygen vacancies and lower the effective cation concentration. These effects contribute to lattice expansion while preserving overall structural symmetry [16,17,18,27,28,29,30].

The hopping length (d) for A (d_A_) and B (d_B_) sites was calculated according to Equations (7) and (8) [26,27]:

(7) $$d_{A}=0.25\cdot a\sqrt{3}$$

(8) $$d_{B}=0.25\cdot a\sqrt{2}$$

The d_A_ and d_B_ are closely related to the a, primarily due to the larger ionic radius of the Zn^2+^ ions compared to the Fe^3+^ ions, which induce lattice expansion. Higher d_A_ and d_B_ values imply increased separation between cations, which raises the energy barrier for charge carrier transfer between sites, thereby impacting electronic and magnetic behavior [16,17,18,27,28,29,30].

### 3.4. BET Analysis

The BET measurements did not allow the determination of the SSA of the samples annealed at 1000 °C. One possible explanation is that the stable form of the SiO_2_ matrix, formed after annealing at 1000 °C, has been reported to exhibit an SSA value below 0.5 m^2^/g, which is below the detection limit of the method used in this study. The SSA values vary in the following order: 375 m^2^/g (SiO_2_) > 245 cm^2^/g ((ZnFe_2_O_4_)_0.5_/(SiO_2_)_0.5_) > 37.5 m^2^/g (ZnFe_2_O_4_) at 400 °C and 215 m^2^/g (SiO_2_) > 115 m^2^/g ((ZnFe_2_O_4_)_0.5_/(SiO_2_)_0.5_) (ZnFe_2_O_4_ SSA value < 0.5 m^2^/g) at 700 °C. The observed SSA values can be explained by the porous SiO_2_ matrix, which not only contributes to the high SSA but also disperses and stabilizes the ferrite nanoparticles, thereby preventing agglomeration [20]. This behavior is typical of oxide materials and aligns with the XRD and AFM results, where higher crystallinity and larger particle sizes result in fewer interparticle pores and alterations in the crystalline phases [20].

### 3.5. AFM Analysis

**Figure 5:**
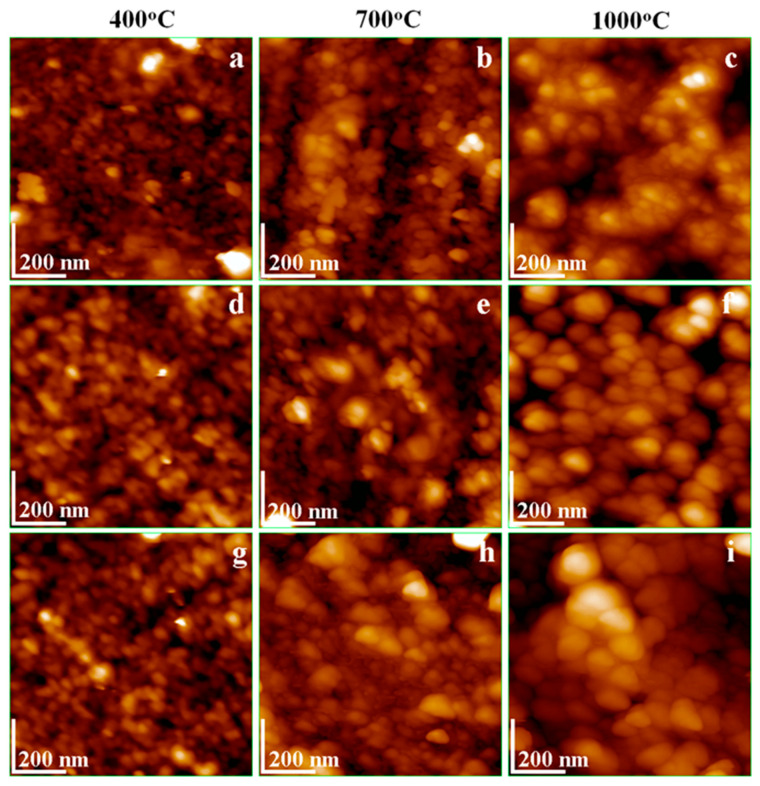
AFM topographic images of the samples: ZnFe_2_O_4_ annealed at (**a**) 400 °C, (**b**) 700 °C, and (**c**) 1000 °C; SiO_2_ annealed at (**d**) 400 °C, (**e**) 700 °C, and (**f**) 1000 °C; and (ZnFe_2_O_4_)_0.5_/(SiO_2_)_0.5_ annealed at (**g**) 400 °C, (**h**) 700 °C, and (**i**) 1000 °C.

Figure 5a shows the morphology of ZnFe_2_O_4_ nanoparticles annealed at 400 °C, revealing predominantly spherical particles with an average diameter of 35 nm uniformly dispersed onto the glass substrate. This uniform adsorption promotes a high degree of particle individualization, minimizing their interactions at the nanoscale. However, small nanoparticles still tend to coalesce, forming local clusters of around 100–150 nm, primarily located at the edges of the image. Increasing the annealing temperature to 700 °C activates fusion between smaller nanoparticles, resulting in a particle size of around 52 nm. The growth of the ferrite crystallite induces slightly squared shapes of the particles in good agreement with the observation in the literature [31]. Their size is very close to that resulting from XRD calculations, proving that these nanoparticles are identical to the observed crystallites. Although the adsorption of the nanoparticles is slightly irregular due to a tendency towards local self-assembly, the particles appear more individualized, and the clustering tendency is significantly reduced. At an annealing temperature of 1000 °C, a well-developed ferrite is observed, imparting a square morphology to the nanoparticles. They resemble small rectangles with rounded edges (Figure 5c). The particle size increases further to 72 nm (in good agreement with XRD results, proving their identification with the observed crystallites), and the nanoparticles are arranged in well-defined positions within the self-assembled thin film.

After annealing at 400 °C, SiO_2_ facilitates the formation of well-individualized, rounded nanoparticles with diameters of about 40 nm. These particles adsorb uniformly onto the glass slide without exhibiting any noticeable clustering tendency (Figure 5d). Upon increasing the annealing temperature to 700 °C, lateral diffusion among smaller nanoparticles leads to the formation of larger aggregates, as shown in Figure 5e. The nanoparticles’ diameter increases to 58 nm, with several clusters measuring approximately 80 nm clearly visible near the center of the image. These nanoparticles generally retain a rounded morphology because of the amorphous silica clustering. Further annealing at 1000 °C results in a significant increase in particle size to approximately 84 nm. The rounded forms become boulder like (partly elongated with deeply rounded sides), as is typical for the natural form of the silica sand found in various technical applications [32,33,34]. They are highly individualized and uniformly adsorbed across the glass substrate (Figure 5f). The behavior of the (ZnFe_2_O_4_)_0.5_/(SiO_2_)_0.5_ nanocomposite at a low annealing temperature closely resembles that of pure SiO_2_, as shown in Figure 5g. At this stage, the nanoparticles are rounded, well-individualized, and uniformly adsorbed, forming a continuous thin film with an average diameter of approximately 55 nm. Increasing the annealing temperature to 700 °C promotes significant development of the ferrite core, in good agreement with XRD data, generating well-developed nanoparticles featuring a distinct square-shaped core with rounded margins and an average size of 65 nm. XRD shows that the ferrite crystallite has a diameter of about 54 nm; the difference to the particle diameter of 65 nm is given by the amorphous SiO_2_ glaze forming a thin crust over the ferrite crystallite. Further annealing at 1000 °C causes the nanoparticles to grow, reaching an average size of 98 nm. Their growth is related to the prominent development of the ferrite core having a diameter of about 79 nm, as observed by XRD, and the outer amorphous SiO_2_ layer leads to a final particle diameter of about 98 nm. They remain well-individualized and exhibit a relatively square morphology with rounded edges, indicative of a strongly developed ferrite phase. The SiO_2_ crust acts as a barrier to the ferrite core’s fusion into larger clusters, allowing a better distribution of magnetic centers within the composite powder.

AFM images (Figure 5) confirm that the nanoparticle size increases with annealing temperature and composition, progressing from ZnFe_2_O_4_ to SiO_2_. This trend is consistent with previous reports on ferrite nanoparticle behavior [32,33]. Notably, the amorphous SiO_2_ coating acts as a physical barrier, effectively isolating the ferrite crystallites and inhibiting the formation of larger clusters. As a result, it promotes the development of well-dispersed and uniformly distributed nanoparticles [34,35].

**Figure 6:**
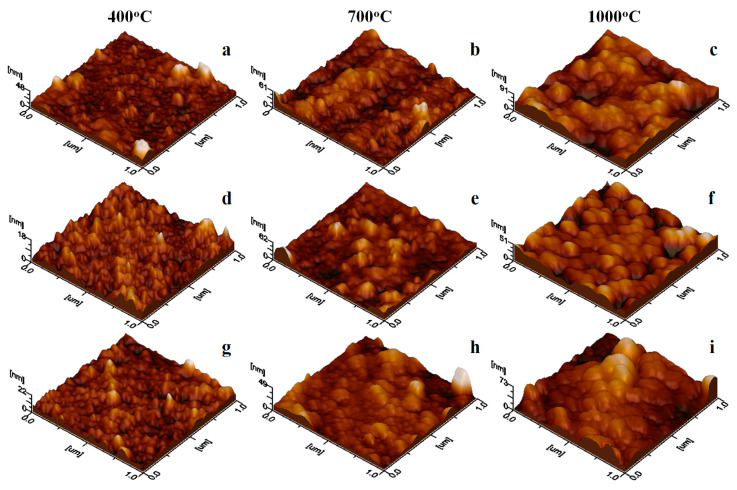
Three-dimensional profiles of the samples: ZnFe_2_O_4_ annealed at (**a**) 400 °C, (**b**) 700 °C, and (**c**) 1000 °C; SiO_2_ annealed at (**d**) 400 °C, (**e**) 700 °C, and (**f**) 1000 °C; and (ZnFe_2_O_4_)_0.5_/(SiO_2_)_0.5_ annealed at (**g**) 400 °C, (**h**) 700 °C, and (**i**) 1000 °C.

The topographical aspects are more clearly observed in the three-dimensional profiles presented in Figure 6.

The uniformly adsorbed small nanoparticles, such as SiO_2_ and (ZnFe_2_O_4_)_0.5_/(SiO_2_)_0.5_ annealed at 400 °C (Figure 6d,g), form smooth thin films, with surface roughness ranging from 3.15 to 5.10 nm, as reported in Table 2. In contrast, the nanoclusters present in the ZnFe_2_O_4_ sample annealed at 400 °C (Figure 6a) lead to an increased surface roughness of 4.01 nm due to ferrite crystallite coalescence, which induces protrusions causing localized height variations. Thus, amorphous SiO_2_ acts as a refining agent of nanostructure. Surface roughness further increases progressively with the development of larger, well-individualized nanoparticles formed after annealing at 700 and 1000 °C. Thus, the (ZnFe_2_O_4_)_0.5_/(SiO_2_)_0.5_ thin film exhibits the highest roughness, as observed in Figure 6i. This variation in surface roughness plays a critical role in the performance of ferrite thin films in technological applications, including sustainable energy and biomechanical energy conversion [7,33].

### 3.6. SEM Analysis

**Figure 7:**
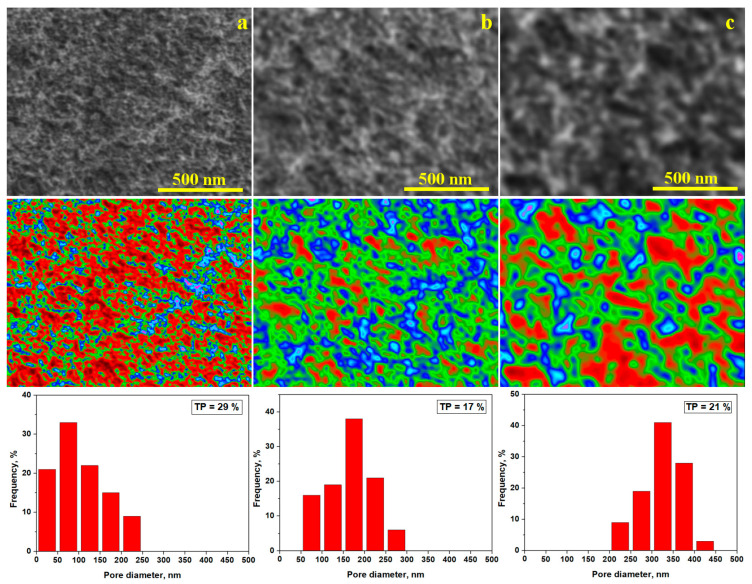
SEM images with pore distribution analysis for the samples annealed at 1000 °C: (**a**) ZnFe_2_O_4_, (**b**); SiO_2_, and (**c**) (ZnFe_2_O_4_)_0.5_/(SiO_2_)_0.5_. Red areas correspond to pores, green areas correspond to well dispersed particles, and blue areas belong to clusters.

Powders annealed at 1000 °C feature the best development of ferrite crystallites and, thus, are most important for magnetic applications. ZnFe_2_O_4_ particles are predominantly small, in good agreement with AFM observations. The absence of insulation material predisposes the ferrite particles to agglomeration due to interparticle attraction forces (Figure 7a). Thus, white clusters are distributed over the surface surrounded by finer ferrite particles. Their square-based crystallites and particle shapes give angular interfaces, resulting in randomly distribute polyhedral pores. The result is that the total porosity of ferrite powder is about 29%, which is in good agreement with the XRD. The microstructural porosity is slightly higher than the one calculated from XRD because of the random disposal of the particles regarding the spreading on the sample holder during the SEM investigation. The mean pore size class is situated in the range of 50–100 nm, as observed in the distribution histogram. Larger pores are completely absent.

The SiO_2_ sample is characterized predominantly by rounded particles, with the shape resembling river gravel. Thus, their connectivity is lower than in pure ferrite allowing for more uniform dispersion on the sample holder (Figure 7b). The mutual tangency points between amorphous SiO_2_ particles ensure a curvy interface generating dendritic pores detected by Image J software. The clusters are less aggregated than in ferrite but larger in size (about 200 nm), and the pores’ dominant size class ranges between 150 and 200 nm, depending on the uniform distribution of the clusters. Blue and green areas dominate the microstructure, and thus, resulting in a total porosity of 17%.

The amorphous SiO_2_ coating formed upon the ferrite core within the (ZnFe_2_O_4_)_0.5_/(SiO_2_)_0.5_ composite prevents agglomeration, which are relatively sparse but with larger sizes of about 250–300 µm (Figure 7b). The particles are well-dispersed on the specimen holder, ensuring a proper interface between them, but the squared shape of the ferrite core influences the disposed angle, increasing the pore sizes. The mean representative pore class is 300–350 µm, which is partially dependent on both cluster size and the spatial arrangement of the individual particles. The squared corner of individualized particles stacks over the rounded side of the clusters, enhancing microstructural pore development. Thus, the total porosity is 21%, which is slightly larger than the theoretical resulting from the XRD calculations. This can be explained by the interaction between the different geometries of the well individualized particles and microstructural clusters.

**Figure 8:**
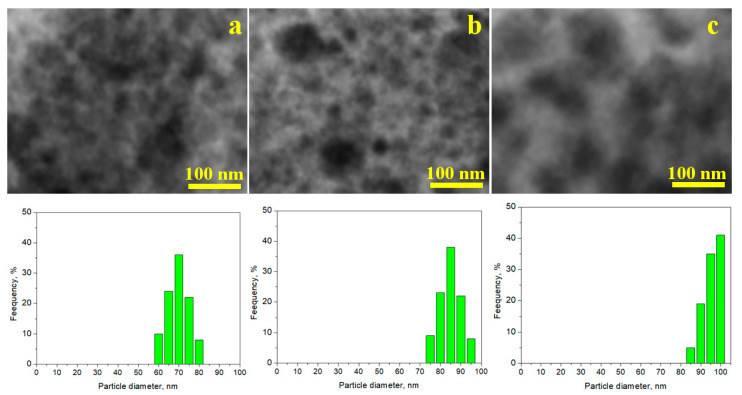
TEM images with particle size distribution analysis for the samples annealed at 1000 °C: (**a**) ZnFe_2_O_4_, (**b**); SiO_2_, and (**c**) (ZnFe_2_O_4_)_0.5_/(SiO_2_)_0.5_.

The samples were randomly dispersed on the TEM grid, to ensure a representative imaging. Ferrite particles and the ferrite core within the composite appear in regions of intense dark contrast, while amorphous SiO_2_ particles and coating surrounding of the composite particles appear in intense grey contrast. Pure ferrite particles (Figure 8a) are well individualized with a high coalescence tendency. The particle size distribution analysis reveals a mean size of 70 nm. Both clusters and well individualized particles of amorphous SiO_2_ are observed in Figure 8b. The particle size distribution reveals a mean value of 85 nm, which is comparable to that of the composite particles being situated at 100 nm, consistent with the AFM measurements.

### 3.7. VSM Analysis

**Figure 9:**
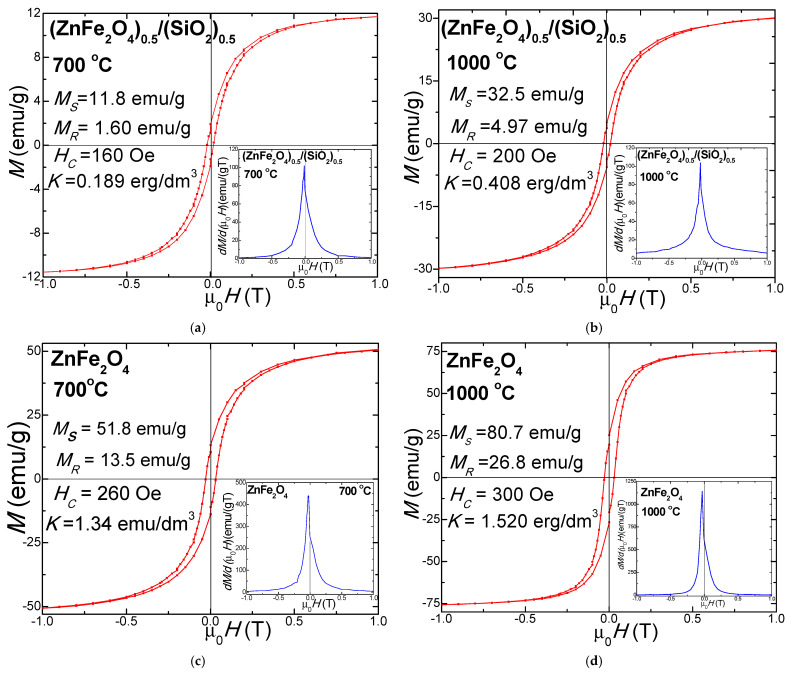
Magnetic hysteresis loops (red) and magnetization derivative (blue) of ZnFe_2_O_4_ and ZnFe_2_O_4_)_0.5_/(SiO_2_)_0.5_ SiO_2_ annealed at 700 ° C (**a**,**c**) and 1000 ° C (**b**,**d**).

The hysteresis loops of (ZnFe_2_O_4_)_0.5_/(SiO_2_)_0.5_ and pure ZnFe_2_O_4_ annealed at 700 °C and 1000 °C are shown in Figure 9. The key magnetic parameters, including saturation magnetization (*M_S_*), remanent magnetization (*M_R_*), coercivity (*H_c_*), and crystalline anisotropy constant (K), were extracted from these curves. The results reveal notable differences between the pure and embedded ZnFe_2_O_4_, highlighting the influence of the non-magnetic matrix on the overall magnetic behavior. These differences further emphasize that the magnetic properties of the nanoparticles are strongly dependent on their crystallinity and morphology [1,11]. Smaller nanoparticles typically exhibit superparamagnetic behavior, as thermal energy is sufficient to overcome their magnetic anisotropy energy barriers. In contrast, larger particles, particularly those with a higher Zn^2+^ content in the spinel lattice, tend to display ferromagnetic behavior, whereas bulk ZnFe_2_O_4_ remains paramagnetic at room temperature [1,11]. The ZnFe_2_O_4_ nanoparticles embedded in the SiO_2_ matrix demonstrate reduced magnetic properties compared to uncoated ZnFe_2_O_4_ reported in previous studies. This attenuation is primarily attributed to an increased surface defect density and magnetic dilution effects, both of which are influenced by the synthesis conditions and the presence of the non-magnetic SiO_2_ layer [1,11].

The coercivity (*H_C_*) of (ZnFe_2_O_4_)_0.5_/(SiO_2_)_0.5_ increases from 160 Oe at 700 °C to 200 Oe at 1000 °C, while in uncoated ZnFe_2_O_4_, *H_C_* rises from 260 Oe to 300 Oe over the same temperature range [1,11]. The *H_c_* of ferrite nanoparticles is governed by a combination of factors, including particle size and shape distribution, magnetocrystalline anisotropy (*K*), internal strain, and interparticle magnetic interactions [1,11]. The lowest *H_C_* values observed for the ZnFe_2_O_4_ embedded in the SiO_2_ matrix at 700 °C are attributed to smaller crystallite sizes and enhanced magnetic disorder. These conditions promote superparamagnetic behavior and result in partial loss of *M_S_* in the absence of an external field [1,11]. The highest *H_C_* values observed in bulk ZnFe_2_O_4_ at elevated annealing temperatures suggest a reduction in both structural and magnetic disorders. In this case, thermal energy appears insufficient to activate the magnetic domain wall motion, thereby limiting magnetization reversal under an applied magnetic field and promoting improved alignment of magnetic moments. Furthermore, low *Hc* values may indicate crystallite coalescence into larger nanostructures, which enhances magnetic coupling and contributes to the observed increase in *M_S_*. Additionally, surface strain induced by the SiO_2_ matrix can reduce particle size and hinder the rotation of both magnetic particles and their surface magnetic moments, further influencing *H_C_* [1,11].

The saturation magnetization (*M_S_*) of (ZnFe_2_O_4_)_0.5_/(SiO_2_)_0.5_ increases from 11.8 emu/g at 700 °C to 32.5 emu/g at 1000 °C, while bulk ZnFe_2_O_4_ shows a corresponding increase from 51.8 emu/g to 80.7 emu/g. This enhancement with increasing annealing temperature is primarily due to the growth in crystallite size and reduction in lattice defects, which facilitate a more effective magnetic moment alignment [1,11,14]. Additionally, changes in the *M_S_* are affected by the formation of antisite defects, which affect the magnetic response independently of the applied magnetic field direction [1,11,36,37]. The significantly lower *M_S_* values observed in the embedded ZnFe_2_O_4_ are due to a higher surface defect density and the magnetic dilution caused by the presence of the non-magnetic matrix [1,11,37]. According to the Néel model of ferrimagnetism, in bulk ZnFe_2_O_4_ spinel, half of the Fe^3+^ ions occupy the A-sites, while the remaining Fe^3+^ ions and the non-magnetic Zn^2+^ ions reside in the B-sites. The magnetic moments of the Fe^3+^ ions at the A-sites are aligned antiparallel to those at the B sites, resulting in partial cancellation and a reduced net magnetic moment [1,11,37,38]. However, at the nanoscale, cation redistribution and thermal effects during annealing can induce a net magnetic moment by altering site occupancy [37]. Additionally, magnetic moment suppression and reduced *M_S_* can occur due to cation disorder and weakened superexchange interactions between A- and B-sites in the spinel structure. In addition to magnetic dilution, the presence of the SiO_2_ matrix introduces surface disorder and increases the density of broken chemical bonds on the particle surfaces. These defects promote spin canting, which disrupts magnetic alignment and further influences the particle size, ultimately affecting the overall magnitude of the *M_S_* [1,11,37,38].

The remanent magnetization (*M_R_*) of (ZnFe_2_O_4_)_0.5_/(SiO_2_)_0.5_ increases from 1.60 emu/g at 700 °C to 4.97 emu/g at 1000 °C, while for bulk ZnFe_2_O_4_, *M_R_* rises from 13.5 emu/g to 26.8 emu/g over the same temperature range [1,11]. The low *M_R_* values observed in the embedded ZnFe_2_O_4_ are indicative of superparamagnetic behavior, where thermal fluctuations prevent stable magnetic alignment in the absence of an external magnetic field [1,11]. The observed increase in *M_R_* with higher annealing temperature is attributed to crystal growth and a reduction in magnetic disorder, leading to enhanced magnetic moment retention [1,11,14].

The crystalline anisotropy constant (*K*) of (ZnFe_2_O_4_)_0.5_/(SiO_2_)_0.5_ rises from 0.189 erg/dm^3^ at 700 °C to 0.408 erg/dm^3^ at 1000 °C, while for non-embedded ZnFe_2_O_4_, *K* rises from 1.34 erg/dm^3^ to 1.520 erg/dm^3^ over the same temperature range [1,11]. Higher K values are typically associated with larger particle sizes and are influenced by factors such as particle shape, crystalline symmetry, and the surface distribution of magnetic ions. These factors collectively determine the preferred magnetization direction along specific crystallographic axes [1,11]. The lower *K* value observed in the embedded sample is attributed to the dilution effect imposed by the non-magnetic SiO_2_ matrix. The increase in *K* with annealing temperature is linked to enhanced crystallinity and a reduction in structural defects [1,11]. Magnetic anisotropy serves as an energy barrier that resists changes in the direction of magnetization, making it a critical parameter in the superparamagnetic behavior of nanoparticles [1,11,37,38]. When thermal energy becomes comparable to or exceeds the anisotropy energy, it can induce magnetization reversal, thereby promoting superparamagnetic behavior [1,11,37,38].

### 3.8. Photocatalytic Analysis

**Figure 10:**
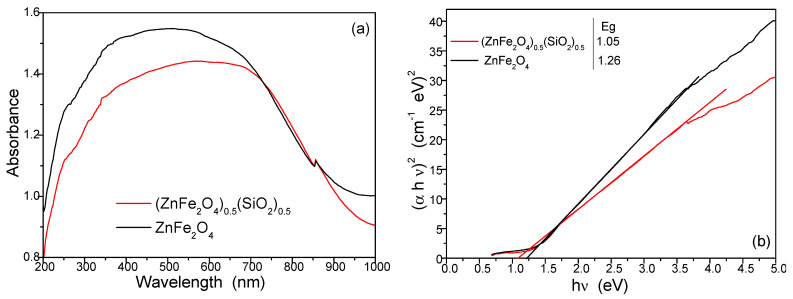
(**a**) UV–Vis absorbance spectra and (**b**) Tauc’s plot of ZnFe_2_O_4_, (ZnFe_2_O_4_)_0.5_/(SiO_2_)_0.5_, and SiO_2_ annealed at 1000 ° C.

The optical properties of the samples annealed at 1000 °C were characterized using UV–Vis spectroscopy (Figure 10a). The optical response of nanoparticles is governed by factors such as quantum confinement, lattice strain, structural defects, impurities, surface effects, the light source, the separation efficiency of electron–hole pairs, the surface structure of the catalyst, microstructure, component characterization, phase structure, and the reaction conditions [21,28,39,40,41,42,43,44,45,46,47,48,49,50,51,52,53,54,55]. A pronounced absorption peak in the visible region indicates promising visible-light photocatalytic activity. The active species verification experiments showed that the hole, hydroxyl radical, and superoxide radical were the main active species in the degradation of the photocatalyst [28,31].

Photocatalytic activity is strongly influenced by the band gap energy, estimated here via Tauc’s method considering the UV–Vis absorption spectra. Figure 10b shows the Tauc plots for samples annealed at 1000 °C. The sample embedded in the SiO_2_ matrix exhibits a reduced band gap of 1.05 eV compared to 1.26 eV for the unembedded samples, attributable to their higher ferrite content. The prominent absorption arises from electron excitation across the band gap, primarily involving transitions from the valence band to the conduction band. Variations in the absorption edge are linked to interface and point defects as well as interactions involving photogenerated electrons [39,40,41,42,43,44,45,46,47,48,49,50,51,52,53].

The photocatalytic process of RhB was analyzed using the first-order kinetic model based on its absorbance (Equation (9)) [21,28,39,40,41,42,43,44,45,46,47,48,49,50,51,52,53]:

(9) $$-ln\frac{A_{t}}{A_{0}^{*}x}=\;k_{i\;}*\;t,$$

where A_t_ is the absorbance of RhB at time t, $A_{0}^{*}$ is the absorbance of RhB after dark adsorption, t is the irradiation time, and k is the apparent kinetic constant.

**Figure 11:**
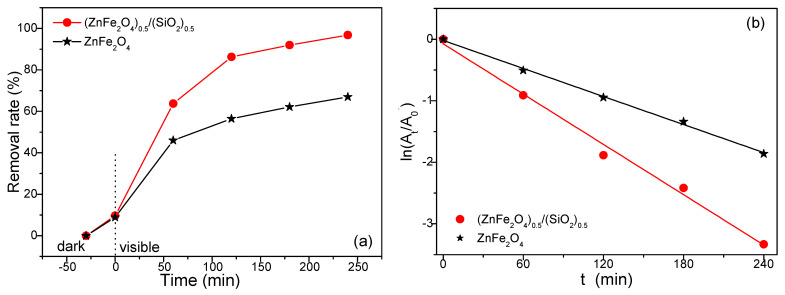
Removal rate of RhB under visible-light irradiation (**a**) and photodegradation kinetic of ZnFe_2_O_4_, (ZnFe_2_O_4_)_0.5_/(SiO_2_)_0.5_, and SiO_2_ annealed at 1000 °C (**b**).

The removal rate of the RhB solution in the case of all the samples is illustrated in Figure 9a. The removal rate involves both adsorption and photocatalysis processes. The existence of ZnFe_2_O_4_ in the SiO_2_ matrix enhances the adsorption capacity of the samples. After 240 min of irradiation, Rhodamine B degradation was 98% ((ZnFe_2_O_4_)_0.5_(SiO_2_)_0.5_) and 64% (ZnFe_2_O_4_), respectively. The photodegradation process is well described by a pseudo-first-order kinetic model and varies linearly with time t (Figure 11b). The linear dependence of photocatalytic degradation on irradiation time is illustrated in Figure 11b.

Upon light irradiation, electron–hole pairs are generated, initiating the photocatalytic process. Suppressing charge recombination allows the excited electrons in the conduction band to reduce surface-adsorbed O_2_ molecules, forming superoxide radicals. Meanwhile, the holes in the valence band oxidize water or hydroxyl groups, producing hydroxyl radicals. The apparent first-order rate constants (*k*) are summarized in Table 3. Based on the *k* values, the photocatalytic activity follows the order (ZnFe_2_O_4_)_0.5_(SiO_2_)_0_._5_ (*k* = 14.1 × 10^−3^ min^−1^, *R*^2^ = 0.9991) > ZnFe_2_O_4_ (*k* = 4.12 × 10^−3^ min^−1^, *R*^2^ = 0.9033), as compared to previously reported data (Table 2). The enhanced photocatalytic performance observed for both (ZnFe_2_O_4_)_0.5_(SiO_2_)_0.5_ and ZnFe_2_O_4_ is primarily attributed to their mesoporous structure and the effective separation of photogenerated electron–hole pairs.

Analysis of the obtained values shows that (ZnFe_2_O_4_)_0.5_(SiO_2_)_0.5_ exhibits the highest photocatalytic activity, with a constant approximatively five times greater than that of ZnFe_2_O_4_. The photocatalytic performance of (ZnFe_2_O_4_)_0.5_(SiO_2_)_0.5_ annealed at 1000 °C is comparable to that of ZnFe_2_O_4_ and other Zn-based ferrite systems reported in previous literature (Table 3).

**Figure 12:**
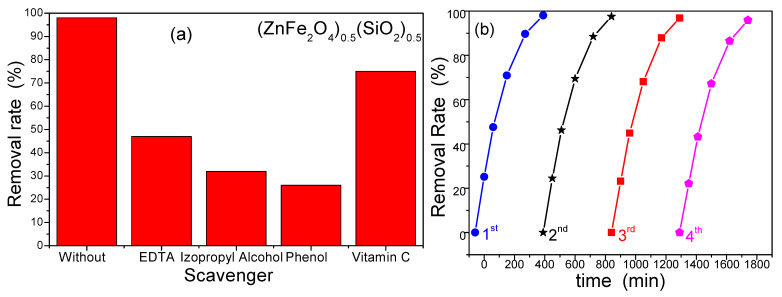
The effect of scavengers on the RhB degradation (**a**) and the reusability of the (ZnFe_2_O_4_)_0.5_(SiO_2_)_0.5_ nanocomposite used in the removal experiments (**b**).

Scavenger experiments were conducted to identify the reactive species involved in RhB degradation. To selectively reduce holes and hydroxyl and superoxide radicals, 5 mM solutions of ethylenediaminetetraacetic acid (EDTA), isopropyl alcohol, phenol, and vitamin C were added to the initial RhB solution. As shown in Figure 12a, the presence of the scavengers significantly reduced the degradation rate compared to the control group, indicating that all three species are important in the photocatalytic process. The tests were performed using the (ZnFe_2_O_4_)_0.5_(SiO_2_)_0.5_ nanocomposite, which contains ZnFe_2_O_4_ embedded within the SiO_2_ matrix that facilitates charge separation by mediating electron–hole transfer. Additionally, this composite effectively generates reactive oxygen species (ROS) capable of degrading the RhB molecule [28]. To assess photocatalyst stability, the (ZnFe_2_O_4_)_0.5_(SiO_2_)_0.5_ nanocomposite was subjected to four consecutive degradation cycles. After each cycle, the catalyst was magnetically recovered, washed with ethanol and water, and reused. The degradation efficiency remained nearly unchanged over the four cycles, confirming the excellent photostability and reusability of the photocatalyst (Figure 12b).

Based on the results, the photocatalytic mechanism can be attributed to the generation of electron–hole pairs under light irradiation. When recombination is suppressed, the photogenerated electrons in the conduction band reduce the adsorbed oxygen to form superoxide radicals, while holes in the valence band oxidize water to produce hydroxyl radicals [28,50,51,52,53,54,55,56]. These reactive oxygen species degrade organic pollutants such as RhB. The enhanced photocatalytic activity observed for the (ZnFe_2_O_4_)_0.5_(SiO_2_)_0.5_ nanocomposite may result from its improved adsorption capacity and the formation of intermediate ZnFe_2_O_4_ energy levels within the SiO_2_ matrix, which promote charge separation. ZnFe_2_O_4_ domains act as mediators of charge transfer, facilitating efficient generation of ROS and contributing to the high degradation efficiency [52,53,54,55,56]. These results confirm both the efficacy of the synthesized material and the suitability of the sol-gel method for fabricating high-performance photocatalysts.

## 4. Conclusions

This study investigated the synthesis of ZnFe_2_O_4_ via thermal decomposition as well as the preparation of a (ZnFe_2_O_4_)_0.5_(SiO_2_)_0.5_ nanocomposite and pure SiO_2_ via the sol-gel method. Thermal analysis revealed distinct decomposition of the Zn^2+^ and Fe^3+^ succinate precursors, leading to the formation of ZnFe_2_O_4_ both non-embedded and embedded within the SiO_2_ matrix. The highest total mass loss was observed for bulk ZnFe_2_O_4_ (67%), followed by the ZnFe_2_O_4_/SiO_2_ nanocomposite (63%) and pure SiO_2_ (48%). FT-IR spectroscopy confirmed the formation of the SiO_2_ matrix and reaction progress. FT-IR and XRD indicated the formation of a single-phase spinel structure and the successful embedding of ZnFe_2_O_4_ into the SiO_2_ matrix. Crystallite sizes increased with the annealing temperature, ranging from 35 to 79 nm for the nanocomposites and 48 to 95 nm for bulk ZnFe_2_O_4_. Magnetic properties improved with annealing temperature and ZnFe_2_O_4_ content: saturation magnetization (Ms) from 11.8 to 80.7 emu/g, remanent magnetization (Mr) from 1.6 to 26.8 emu/g, coercivity (Hc) from 160 to 300 Oe, and anisotropy constant (K) from 0.189 × 10^−3^ to 1.520 × 10^−3^ erg/cm^3^. These enhancements were attributed to increased particle size, crystallinity, and reduced surface spin disorder. The best photocatalytic performance was recorded for the (ZnFe_2_O_4_)_0.5_(SiO_2_)_0.5_ nanocomposite, which exhibited the best photocatalytic performance, presumably due to the synergistic effect of ZnFe_2_O_4_ energy levels within the silica matrix and an optimized Zn–Fe balance in the ferrite structure. This is likely due to additional energy levels in the conduction band that promote the recombination of photogenerated carriers. Scavenger experiments show that holes and hydroxyl and superoxide radicals are implicated in photocatalytic processes.

## Figures and Tables

**Table 1:** The structural parameters of (ZnFe_2_O_4_)_0.5_/(SiO_2_)_0.5_ and ZnFe_2_O_4_ samples estimated by XRD.

Sample	Temp [°C]	D_CS_, [nm]	a, [Å]	V, [Å^3^]	d_p_ [g/cm^3^]	d_XRD_ [g/cm^3^]	P [%]	d_A_ [nm]	d_B_ [nm]
(ZnFe_2_O_4_)_0.5_/(SiO_2_)_0.5_	400	35	8.345	581	4.554	5.512	17.4	3.613	2.950
	700	54	8.352	583	4.586	5.493	16.5	3.617	2.953
	1000	79	8.367	586	4.625	5.465	15.4	3.623	2.958
ZnFe_2_O_4_	400	32	8.375	587	4.388	5.456	19.6	3.626	2.961
	700	48	8.389	590	4.375	5.428	19.4	3.632	2.966
	1000	69	8.411	595	4.362	5.383	19.0	3.642	2.974
Errors	-	±2.0	±0.015	±1.0	±0.011	±0.016	±1.0	±0.010	±0.010

**Table 2:** ZnFe_2_O_4_, SiO_2_ and (ZnFe_2_O_4_)_0.5_/(SiO_2_)_0.5_ sample parameters measured with AFM.

Sample	Temperature [°C]	Height [nm]	Rq Roughness [nm]	Average Particle Diameter [nm]
ZnFe_2_O_4_	400	48 ± 1.44	4.01 ± 0.24	35 ± 2.1
	700	61 ± 1.83	6.83 ± 0.40	52 ± 3.12
	1000	91 ± 2.73	12.6 ± 0.75	72 ± 4.32
SiO_2_	400	18 ± 0.54	2.04 ± 0.12	40 ± 2.4
	700	62 ± 1.86	5.10 ± 0.30	58 ± 3.48
	1000	51 ± 1.53	7.37 ± 0.44	84 ± 5.04
(ZnFe_2_O_4_)_0.5_/(SiO_2_)_0.5_	400	22 ± 0.66	1.89 ± 0.11	55 ± 3.3
	700	49 ± 1.47	3.15 ± 0.18	65 ± 3.9
	1000	73 ± 2.19	11.2 ± 0.67	98 ± 5.88

**Table 3:** Comparison of first-order rate constants for various ZnFe_2_O_4_ photocatalysts with Rhodamine B under visible light.

Sample	Lights	Dyes	k × 10^−3^	Reference
(ZnFe_2_O_4_)_0.5_(SiO_2_)_0.5_	Visible	Rhodamine B	14.1	This work
ZnFe_2_O_4_/ ZnO	Visible	Rhodamine B	3.5	[36]
Co_0_._6_Zn_0.4_Fe_2_O_4_	Visible	Rhodamine B	15.1	[37]
Co_3_O_4_/ZnFe_2_O_4_	Visible	Rhodamine B	9.32	[38]
ZnFe_2_O_4_/Cd-Se_5_GO	Visible	Rhodamine B	16.40	[54]
ZnS/K_2_S_2_O_8_	Visible	Rhodamine B	11.98	[40]
20%ZnFe_2_O_4_/80%SnO_2_	Visible	Rhodamine B	38	[41]
33.3%ZnFe_2_O_4_/66.7%SnO_2_	Visible	Rhodamine B	33	[41]
THCRT/ ZnFe_2_O_4_/ZnO	Visible	Rhodamine B	32.9	[42]
THCRT/ ZnFe_2_O_4_/ZnO	Visible	Rhodamine B	22.6	[42]
ZnFe_2_O_4_/ZnO-0.1	Visible	Rhodamine B	18.5	[42]
ZnFe_2_O_4_/NaNbO_3_	Visible	Rhodamine B	21.5	[43]
ZnO–ZnFe_2_O_4_	Visible	Rhodamine B	18.5	[44]
ZnO/ZnFe_2_O_4_/zeolite/ PMS	Visible	Rhodamine B	10	[45]
ZnFe_2_O_4_/BiOBr	Visible	Rhodamine B	7.8	[46]
ZnFe_2_O_4_@TiO_2_@Ag_2_O	Visible	Rhodamine B	10.2	[47]

## Data Availability

Data are available on request from the corresponding author.
